# Identification of key pyroptosis-related genes and microimmune environment among peripheral arterial beds in atherosclerotic arteries

**DOI:** 10.1038/s41598-023-50689-x

**Published:** 2024-01-02

**Authors:** Jing-Wen Liu, Zhao-Hua Zhang, Xiao-Shuo Lv, Ming-Yuan Xu, Bin Ni, Bin He, Feng Wang, Jie Chen, Jian-Bin Zhang, Zhi-Dong Ye, Peng Liu, Jian-Yan Wen

**Affiliations:** 1 Peking University China-Japan Friendship School of Clinical Medicine, NO. 2 Yinghua Eastern Road, Beijing, China; 2 Department of Cardiovascular Surgery, China-Japan Friendship Hospital, NO. 2 Yinghua Eastern Road, Beijing, 10029 China; 3 Graduate School of Peking, Union Medical College, No.1 Shuaifuyuan Wangfujing Dongcheng District, Beijing, China

**Keywords:** Computational biology and bioinformatics, Immunology, Cardiovascular biology

## Abstract

Atherosclerosis is a chronic inflammatory disease characterized with innate and adaptive immunity but also involves pyroptosis. Few studies have explored the role of pyroptosis in advanced atherosclerotic plaques from different vascular beds. Here we try to identify the different underlying function of pyroptosis in the progression of atherosclerosis between carotid arteries and femoral. arteries. We extracted gene expression levels from 55 advanced carotid or femoral atherosclerotic plaques. The pyroptosis score of each sample was calculated by single-sample-gene-set enrichment analysis (ssGSEA). We then divided the samples into two clusters: high pyroptosis scores cluster (PyroptosisScoreH cluster) and low pyroptosis scores cluster (PyroptosisScoreL cluster), and assessed functional enrichment and immune cell infiltration in the two clusters. Key pyroptosis related genes were identified by the intersection between results of Cytoscape and LASSO (Least Absolute Shrinkage and Selection Operator) regression analysis. Finally, all key pyroptosis related genes were validated in vitro. We found all but one of the 29 carotid plaque samples belonged to the PyroptosisScoreH cluster and the majority (19 out of 26) of femoral plaques were part of the PyroptosisScoreL cluster. Atheromatous plaque samples in the PyroptosisScoreL cluster had higher proportions of gamma delta T cells, M2 macrophages, myeloid dendritic cells (DCs), and cytotoxic lymphocytes (CTLs), but lower proportions of endothelial cells (ECs). Immune full-activation pathways (e.g., NOD-like receptor signaling pathway and NF-kappa B signaling pathway) were highly enriched in the PyroptosisScoreH cluster. The key pyroptosis related genes GSDMD, CASP1, NLRC4, AIM2, and IL18 were upregulated in advanced carotid atherosclerotic plaques. We concluded that compared to advanced femoral atheromatous plaques, advanced carotid atheromatous plaques were of higher grade of pyroptosis. GSDMD, CASP1, NLRC4, AIM2, and IL18 were the key pyroptosis related genes, which might provide a new sight in the prevention of fatal strokes in advanced carotid atherosclerosis.

## Introduction

Atherosclerosis is a chronic disease with complex pathogenesis that seriously affects the health of middle- and older-aged individuals. Atherosclerosis is characterized by chronic inflammatory lesions of arterial walls caused by a disorder of lipid metabolism^1–3^. Innate immune cells and adaptive immune cells play an important role in the pathogenesis and development of the disease^4^.

There is evidence to suggest that carotid plaques are more unstable and dangerous than femoral plaques in advanced atherosclerosis.^5,6^. Steenman et al. (2018) showed a high prevalence of osteoid metaplasia in femoral arteries (FA) compared to carotid arteries (CA). Consisting with morphological data, genes involved in skeletal (bone) development and muscle function were more strongly expressed in FA than in CA^7^. Besides, Steenman et al. (2018) confirmed that not the cardiovascular risk factors but the arterial beds were significant associations with types of calcifications, which indicated that regional differences may influence the expression of genes and subsequent processes that regulate plaque stability.

Pyroptosis, a recently discovered pro-inflammatory form of cell death distinct from apoptosis and necrosis, may be related to the stability of atherosclerotic plaques^8,9^. Pyroptosis activates a variety of caspases (cysteinyl aspartate specific proteinases) mediated by inflammasomes, including caspase-1, leading to cell lysis and release of a variety of pro-inflammatory factors into the extracellular environment. Atherosclerosis, pyroptosis, and immune infiltration are known to be related to each other^4,10,11^, yet so far, no study has examined whether advanced atherosclerotic plaques from different vascular beds behave the same in regard to these processes. Exploring if and how plaques differ from each other is crucial to understand the process of advanced plaque formation and the causes of serious complications. In this study, we conducted comprehensive computational bioinformatic analysis using the GSE100927 dataset to examine the correlation between pyroptosis-related genes and different immune cells. We used a machine learning method to screen for gene signatures related to plaque pyroptosis in order to reveal the pyroptosis status of atherosclerotic plaques from different vascular beds.

## Materials and methods

### Data acquisition

Before data analysis, the inclusion criteria of sequencing datasets were formulated as follows: 1. To facilitate subsequent data merging, only the mRNA microarray sequencing was included; 2. The sequencing samples included carotid plaques and femoral plaques, and both carotid and femoral samples were described as advanced atheromatous plaques, which were consistent with the AHA grade 4 to 6 published in 1995^12^; 3. Complete mRNA expression matrix files can be obtained. According to this microarray data inclusion criteria, matrix files of GSE100927 were downloaded from the GEO database (http://www.ncbi.nlm.nih.gov/geo/). We extracted 29 atheromatous carotid plaques and 26 atheromatous femoral plaques from the dataset. The dataset was based on GPL17077 (Agilent-039494 SurePrint G3 Human GE v2 8 × 60 K Microarray 039,381).

### Intra-group data repeatability test

We used the Spearman correlation test to assess repeatability among different samples, and the PheatMap package to show correlations between all samples in the dataset. Principal component analysis (PCA) was performed using the ggplot2 package to visualize gene expression and to assess sample relationships and variation.

### Detection of pyroptosis-related genes and evaluation of pyroptosis scores and immune infiltration of atherosclerotic plaques

Pyroptosis-related genes were identified using two methods. First pyroptosis-related genes were identified in the Gene Database (https://www.genecards.org/), and those with relevance scores > 1.5 were selected. Additional genes were identified from literature^13^. A heatmap of pyroptosis-related genes was generated with the Pheatmap R package (version 1.0.12) to visualize gene expression in different samples. Single sample gene set enrichment analysis (ssGSEA) was conducted to calculate pyroptosis scores based on pyroptosis-related genes using the GSVA R package (version 1.42.0)^14^. Samples were then divided into a high pyroptosis score cluster (PyroptosisScoreH cluster) and a low pyroptosis score cluster (PyroptosisScoreL cluster) based on a cutoff value, which was set to the average pyroptosis score of all samples. A PCA plot was used to assess sample variation after regrouping. Immune and stromal scores were calculated using the ESTIMATE R package (version 1.0.13)^15^, using gene expression level data. In order to explore immune infiltration among different atheromatous plaques and the correlation between different types of immune cells, we used the CIBERSORT (https://cibersort.stanford.edu/)^16^ and MCP-counter (http://github.com/ebecht/MCPcounter)^17^ tools. A correlation heatmap was generated using the corrplot package (version 0.90) to visualize the correlations of different types of infiltrating immune cells. A bar graph was plotted using the ggplot2 package (version 3.3.5) to illustrate the different abundances of immune cells in the PyroptosisScoreH cluster and PyroptosisScoreL cluster.

### Functional enrichment analysis and gene set enrichment analysis (GSEA)

Differentially expressed genes (DEGs) in the PyroptosisScoreH cluster and PyroptosisScoreL cluster were identified using the Bioconductor package “limma” (version 3.50.0). The adjusted p-value cutoff was set to 0.05 at a |log2(Fold Change) |> 1. DEGs were used for Gene Ontology (GO), KEGG database^18–20^ (https://www.kegg.jp/) pathway enrichment analysis, and GSEA, which were performed using the “enrichplot” R package (version 1.14.1) and the “clusterProfiler” R package (version 4.2.0)^21^. ssGSEA was performed to evaluate the activity of immune-related pathways. The immune-related pathways gene set (Supplementary Table S1) was obtained from a published review^22^.

### Construction of the protein–protein interaction (PPI) networks and identification of hub genes

We used the STRING database (http://string-db.org/), an online search tool for the retrieval of interacting genes^23^, to construct PPI networks of pyroptosis-related genes with interaction score > 0.7. Cytoscape (http://cytoscape.org/) was used to visualize the PPI network^24^, and the MCODE and CytoHubba plugins were used with default parameters to identify the top ten hub genes.

### LASSO regression analysis for pyroptosis-related signature genes

First, we calculated the correlation between pyroptosis-related genes and pyroptosis by Spearman analysis, and genes with correlation coefficients > 0.5 were screened. We used the identified genes in the LASSO regression (using the glmSparseNet package (version 1.12.0)), an analysis for fitting selected variables in high dimensional generalized linear models^25^. Pyroptosis-related signature genes were identified from the LASSO regression analysis.

### Immunohistochemistry (IHC)

This study was approved by the Medical Ethics Committee of the China-Japan Friendship Hospital of Beijing, China (2019–25-1) and received informed consent from all of the patients. The Declaration of Helsinki was strictly followed. Excluding criterion including patients suffering from non-atherosclerotic peripheral arterial disease, thrombosis or restenosis. Ten carotid and Ten femoral advanced atheromatous plaque samples were collected from in-hospital patients who underwent carotid endarterectomy (CEA) or femoral endarterectomy (FEA). Both samples were dehydrated and embedded in paraffin. Afterward, samples were sequential sliced into 4-μm sections followed by dewaxing and rehydration. Sections with the most severe lesions were then blocked and incubated with different primary antibodies including GSDMD (1:100, ABclonal; A20473), CASP1(1:200, Proteintech; 22915-1-AP), NLRC4(1:100, Abclonal; A13117), AIM2(1:100, Proteintech; 20590-1-AP) and IL18(1:100, ABclonal; A20473) at 4 °C overnight. The total tissue area and integrated optic density (IOD) of sections stained in yellow–brown was calculated through Image-Pro Plus 6.0 software (IPP 6.0, Media Cybernetics, United States). IOD per unit area represented the level of gene expression.

### Western blot analysis (WB)

Total protein in atherosclerotic plaque samples was extracted using the Solarbio protein extraction kit (BC3710); concentrations were quantified by a BCA protein assay kit (LABLEAD, B5000-500T). For immunoblotting, equal amounts of proteins were separated on an 4% to 12% SDS-PAGE gel and electrophoretically transferred to a polyvinylidene difluoride microporous membrane (0.22 μm pore size; Beyotime) and blocked with 5% non-fat milk for 1 h at room temperature, then blotted with primary antibody (Supplementary Table S2) of targeted proteins overnight at 4 ℃. After incubation with HRP-conjugated secondary antibody for 1 h at room temperature, the membranes were developed using the PierceTM ECL Western Blotting Substrate (Thermo Scientific; Cat: 32,209). Quantitative analysis was carried out by Bio‐Rad ChemiDoc XRS image analysis system according band density. Restore Western Blot Stripping Buffer (APPLYGEN; Cat: P1651) was used to strip membranes when it’s needed.

### Statistical analysis

**Figure 1:**
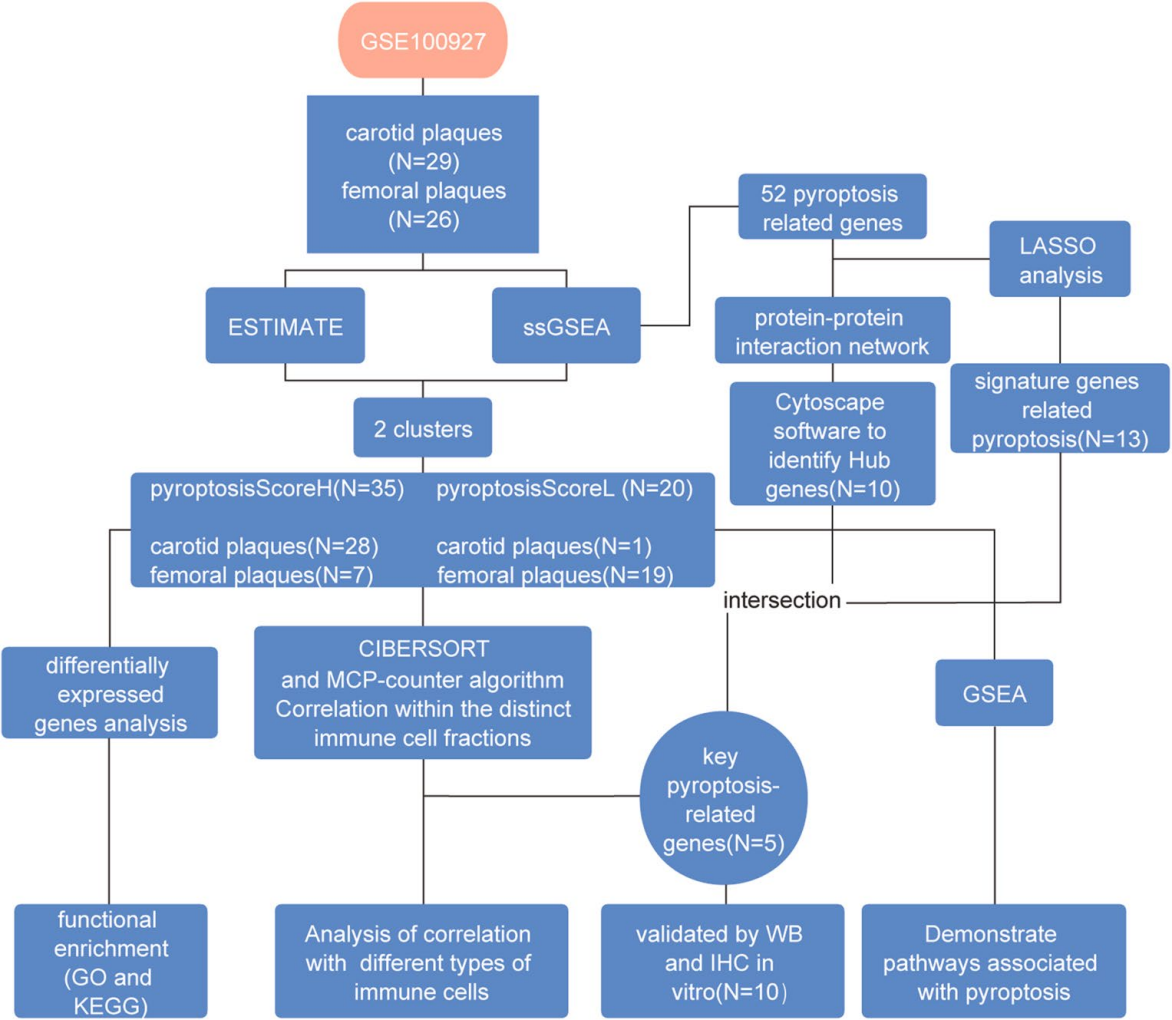
Workflow diagram. Analysis pipeline of expression values from microarrays.

All statistical analyses and graphical visualizations were conducted using R software (version 4.0.1) and GraphPad Prism 8. Categorical variables were presented as counts (proportions), while continuous data were reported as mean (± standard deviation). T-tests were employed to assess significant differences in continuous parameters between two or more groups. The χ^2^ test or Fisher's exact test (when expected frequencies were low) was used for comparing nominal variables among groups. Nonparametric Spearman's rank correlation test was utilized for correlation analysis. In order to control the false discovery rate (FDR), the Benjamini–Hochberg method was used to correct the p value during differential gene analysis and gene enrichment analysis. The FDR less than 0.05 was acceptable. A p-value less than 0.05 (two-sided) was considered statistically significant. (*p <  = 0.05, **p ≤ 0.01, ***p ≤ 0.001, ****p ≤ 0.0001). The overall flow diagram is shown in n Fig. 1.

### Ethics declarations

This study was performed in line with the principles of the Declaration of Helsinki. Approval was granted by the Medical Ethics Committee of the China-Japan Friendship of Beijing, China (2019-25-1), and we received informed consent from all patients. Specimens were handled in accordance with legal and ethical regulations.

## Results

### Checking the quality of GSE100927

In order to assess data quality, we used the Spearman correlation test to calculate the repeatability between different samples, and PCA was performed to assess sample relationships and variation. A heatmap based on the Spearman correlation test shows that samples in the same group are strongly associated with each other (Supplementary Fig. S1a). A PCA of the dataset revealed that intergroup differences are considerably larger than intragroup differences. (Supplementary Fig. S1b).

### Detection of pyroptosis-related genes and evaluation of pyroptosis scores, immune scores, and stromal scores

**Figure 2:**
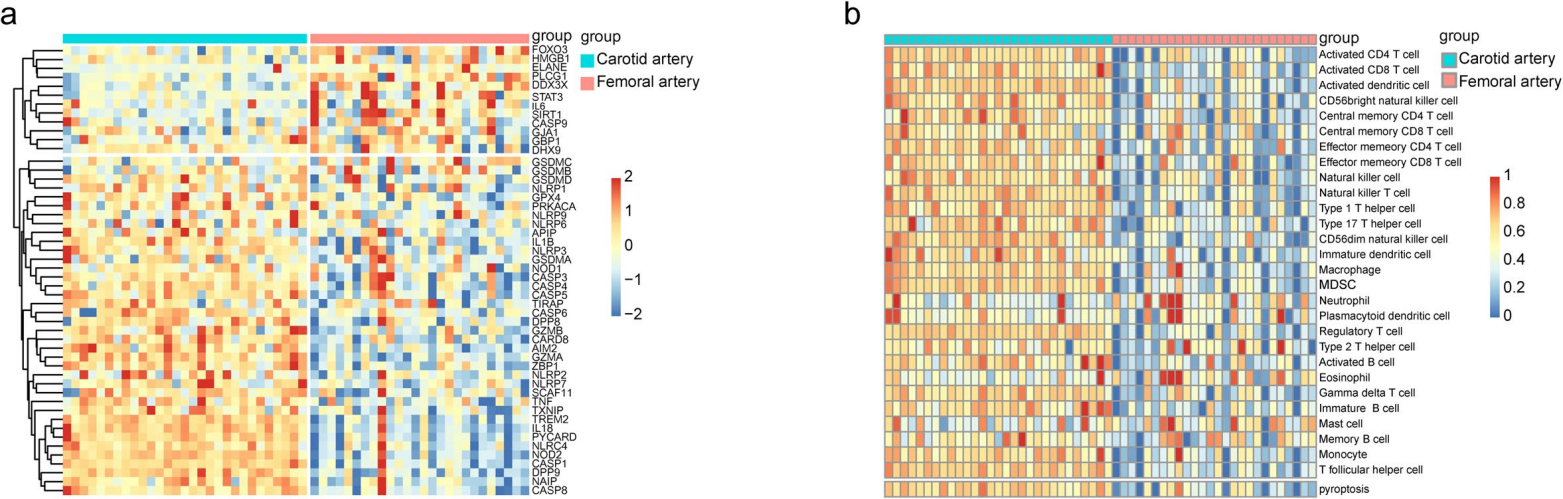
Expression levels of the 50 pyroptosis-related genes and pyroptosis scores of all samples. (**a**) Heatmap (blue: low expression level; red: high expression level) of pyroptosis-related genes in carotid and femoral plaques. (**b**) Heatmap of ssGSEA scores of samples that were clustered based on the Euclidean distance.

**Figure 3:**
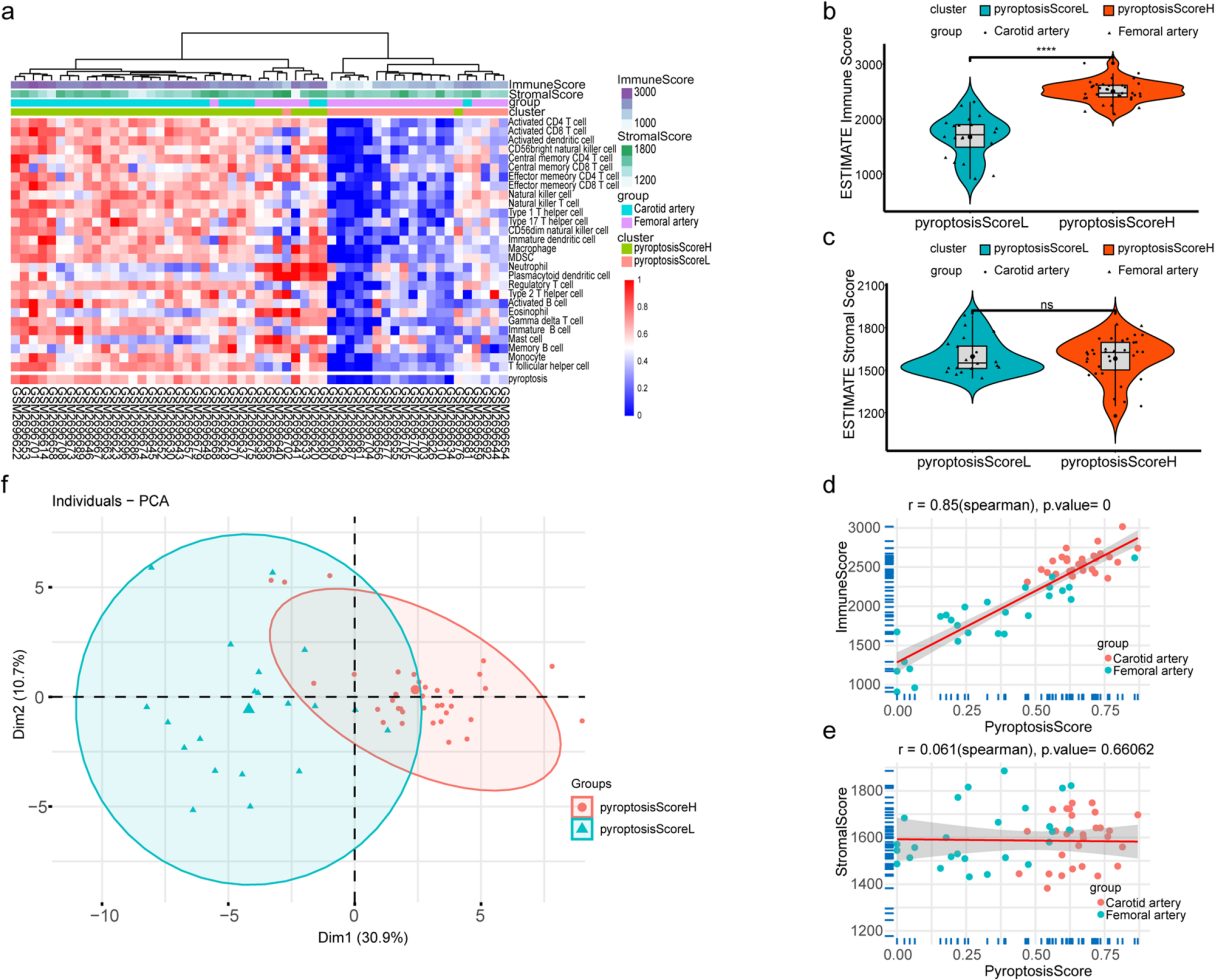
Atheromatous plaque clusters and correlations between pyroptosis scores and immune scores. (**a**) Heatmap of ssGSEA scores of two clusters (PyroptosisScoreH and PyroptosisScoreL) and immune and stromal scores calculated by the ESTIMATE algorithm for all samples. X-label: sample labels. Y-label: 28 types of immune cells and pyroptosis. (**b**,**c**) Violin plots of immune scores and stromal scores in the PyroptosisScoreH cluster and in the PyroptosisScoreL cluster. (**d**,**e**) Scatter plots of the linear fit of pyroptosis score with immune and stromal scores. (**f**) Principal component analysis of the PyroptosisScoreH and PyroptosisScoreL clusters.

We analyzed 52 pyroptosis-related genes (Supplementary Table S3), 50 of which were detected in our gene expression matrix. As shown in Fig. 2a, there are significant differences in pyroptosis-related gene expression between carotid plaques and femoral plaques. Figure 2b shows the enrichment of pyroptosis-related genes in each sample (ssGSEA scores). We chose the average pyroptosis score of all samples as a cutoff value to organize samples into two clusters: those with high pyroptosis scores (PyroptosisScoreH cluster) and those with low pyroptosis scores (PyroptosisScoreL cluster) (Fig. 3a). All but one sample from carotid plaques fell in the PyroptosisScoreH cluster. We then calculated immune and stromal scores using the ESTIMATE algorithm. Only one carotid plaque had a low immune score (Fig. 3a).

We assessed the differences between the two clusters using violin diagrams (Fig. 3b,c). We found that samples in the PyroptosisScoreH cluster had higher immune scores than those in the PyroptosisScoreL cluster (p < 0.05). We subsequently explored the relationship between pyroptosis score and immune and stromal scores using linear fitting. The pyroptosis score is positively correlated with the immune score (R-squared = 0.85, p < 0.001) and not significantly correlated with the stromal score (R-squared = 0.061, p = 0.661) (Fig. 3d,e). PCA results show that the pyroptosis score could be helpful in distinguishing distinct groups of plaque (Fig. 3f).

### Fraction of immune cells and correlation with pyroptosis in atheromatous plaques

**Figure 4:**
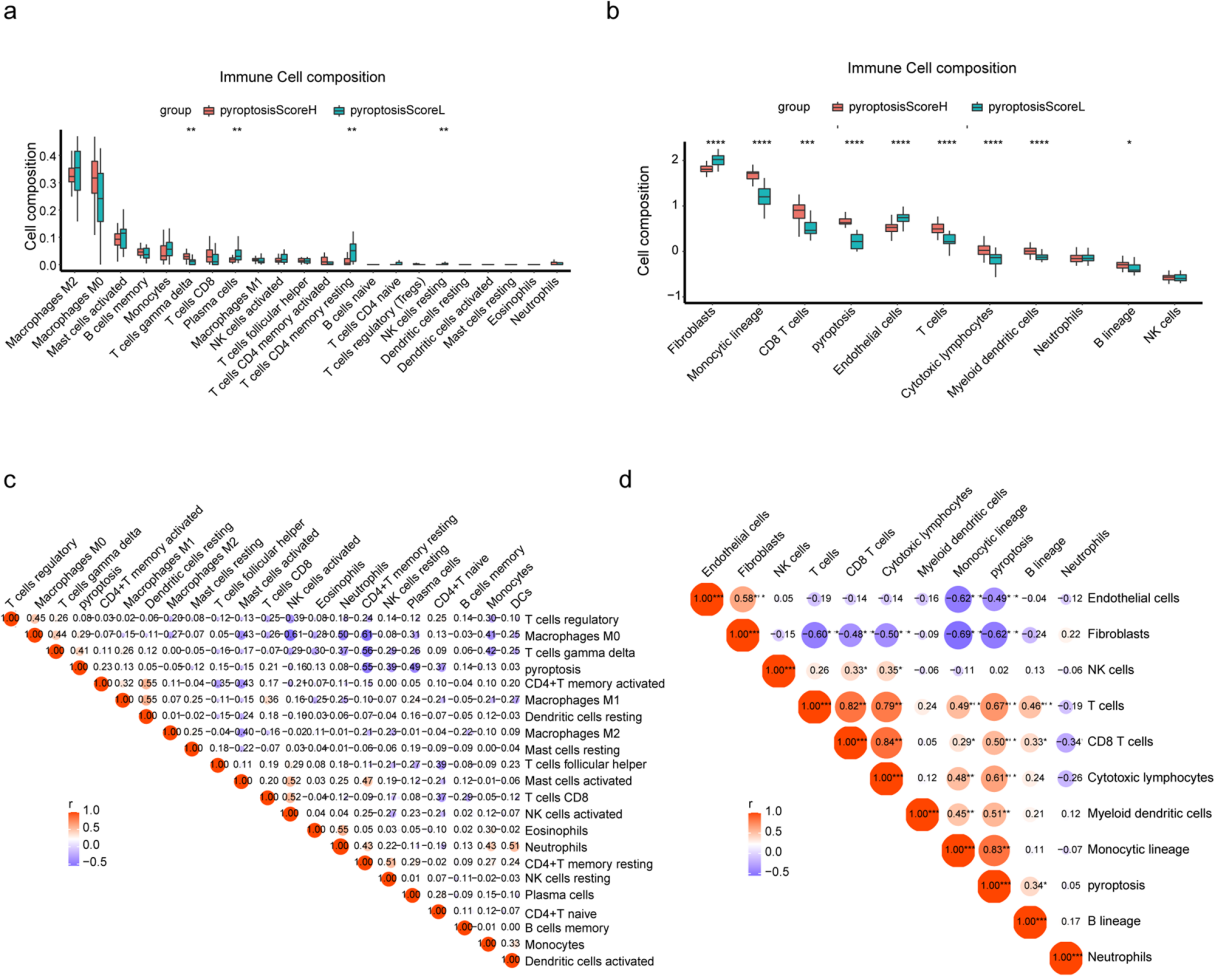
Different algorithms demonstrating the relationship between immune infiltration and pyroptosis. (**a**) Relative abundance of immune cells in different clusters calculated by CIBERSORT. (**b**) Relative abundance of immune cells in different clusters calculated by MCP-Counter. (**c**) Correlation of immune cell types and pyroptosis calculated by CIBERSORT. (**d**) Correlation of immune cell types and pyroptosis calculated by MCP-Counter. (r indicated the correlation coefficient; larger bubbles indicate larger absolute values of correlation coefficient; *p < 0.05, **p < 0.01, ***p < 0.001).

To explore the composition and correlation of immune cells in atheromatous plaques in different states of pyroptosis, we used CIBERSORT and the MCP-counter algorithm. CIBERSORT revealed that atheromatous plaques in the PyroptosisScoreH cluster had a higher proportion of gamma delta T cells, but a lower fraction of plasma cells and CD4 memory resting T cells (Fig. 4a). Besides, the trend in M2 macrophages was upregulation in low pyroptosis group. In addition, pyroptosis was positively correlated with gamma delta T cells (r = 0.41, p < 0.01) (Fig. 4c). Yet all plaques contained mainly macrophages, and there was no difference in this cell population between the two clusters (Supplementary Figs. S2, S3a). We used the MCP-counter algorithm to quantify the absolute abundance of eight immune cell types and two stromal cells, and demonstrated that atheromatous plaques in the PyroptosisScoreH cluster had a higher rate of monocytic lineage, CD8 + T cells, T cells, cytotoxic lymphocytes, and myeloid dendritic cells, but a lower fraction of fibroblasts and endothelial cells (Fig. 4b). In this analysis, pyroptosis was positively correlated with monocytic lineage (r = 0.83, p < 0.001), CD8 + T cells (r = 0.50, p < 0.001), T cells (r = 0.67, p < 0.001), cytotoxic lymphocytes (r = 0.61, p < 0.001), and myeloid dendritic cells (r = 0.51, p < 0.001) and negatively correlated with fibroblasts (r =  − 0.62, p < 0.001) and endothelial cells (r =  − 0.49, p < 0.001) (Fig. 4d).

### Functional analysis based on pyroptosis score groups

**Figure 5:**
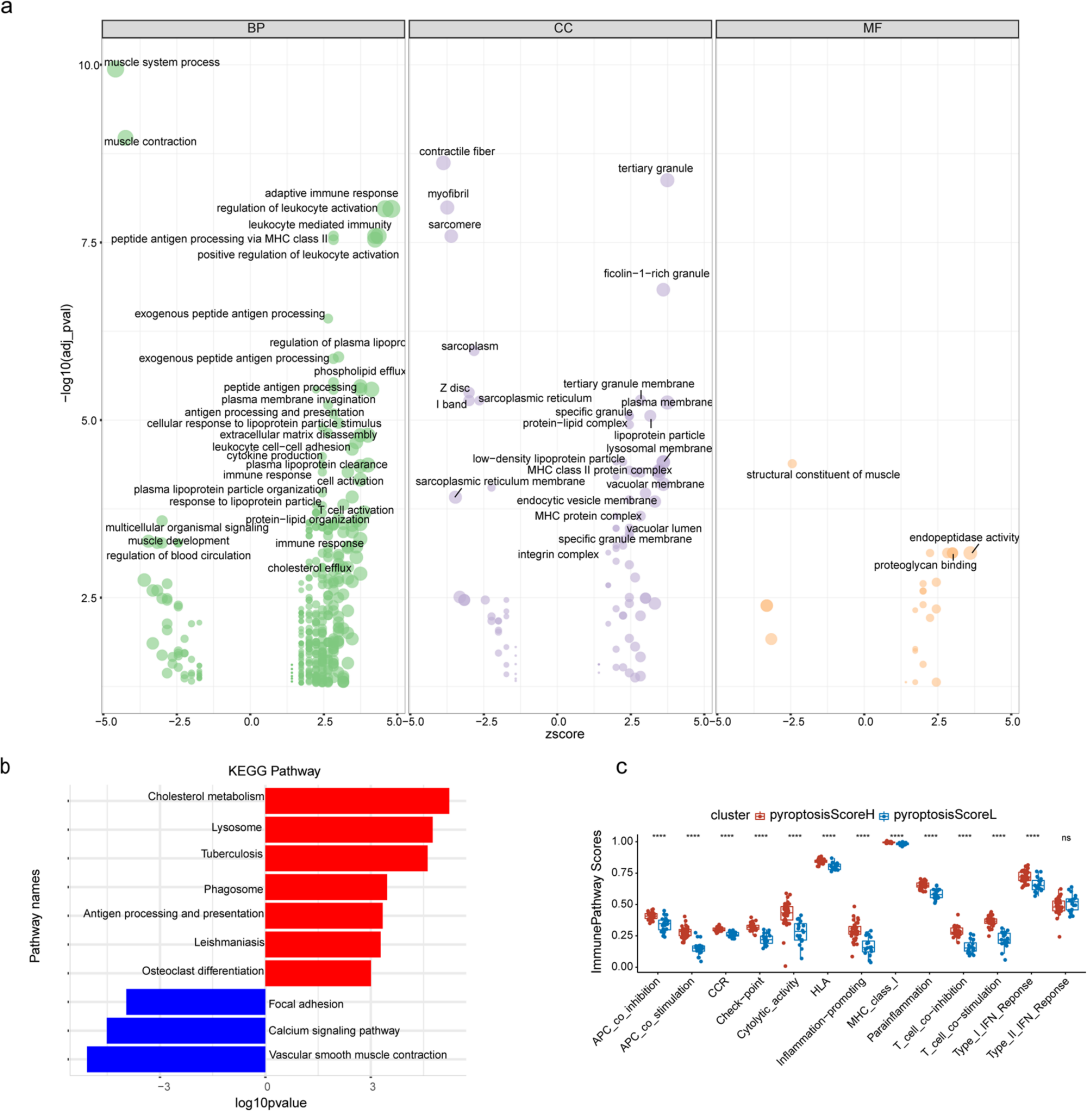
Functional analysis and immune pathways enriched in PyroptosisScoreH and PyroptosisScoreL clusters. (**a**) Bubble graph for GO enrichment (a larger bubble indicates more enriched genes and the z-score reflects the overall expression trend of the genes involved in this function. Z-score > 0: up-regulated; z-score < 0: down-regulated). (**b**) Bar plot for KEGG pathways (a longer bar indicates more enriched genes and more obvious differences. X > 0: up-regulated; X < 0: down-regulated). (**c**) Comparison of the enrichment scores of 13 immune-related pathways between the PyroptosisScoreH and PyroptosisScoreL clusters.

**Figure 6:**
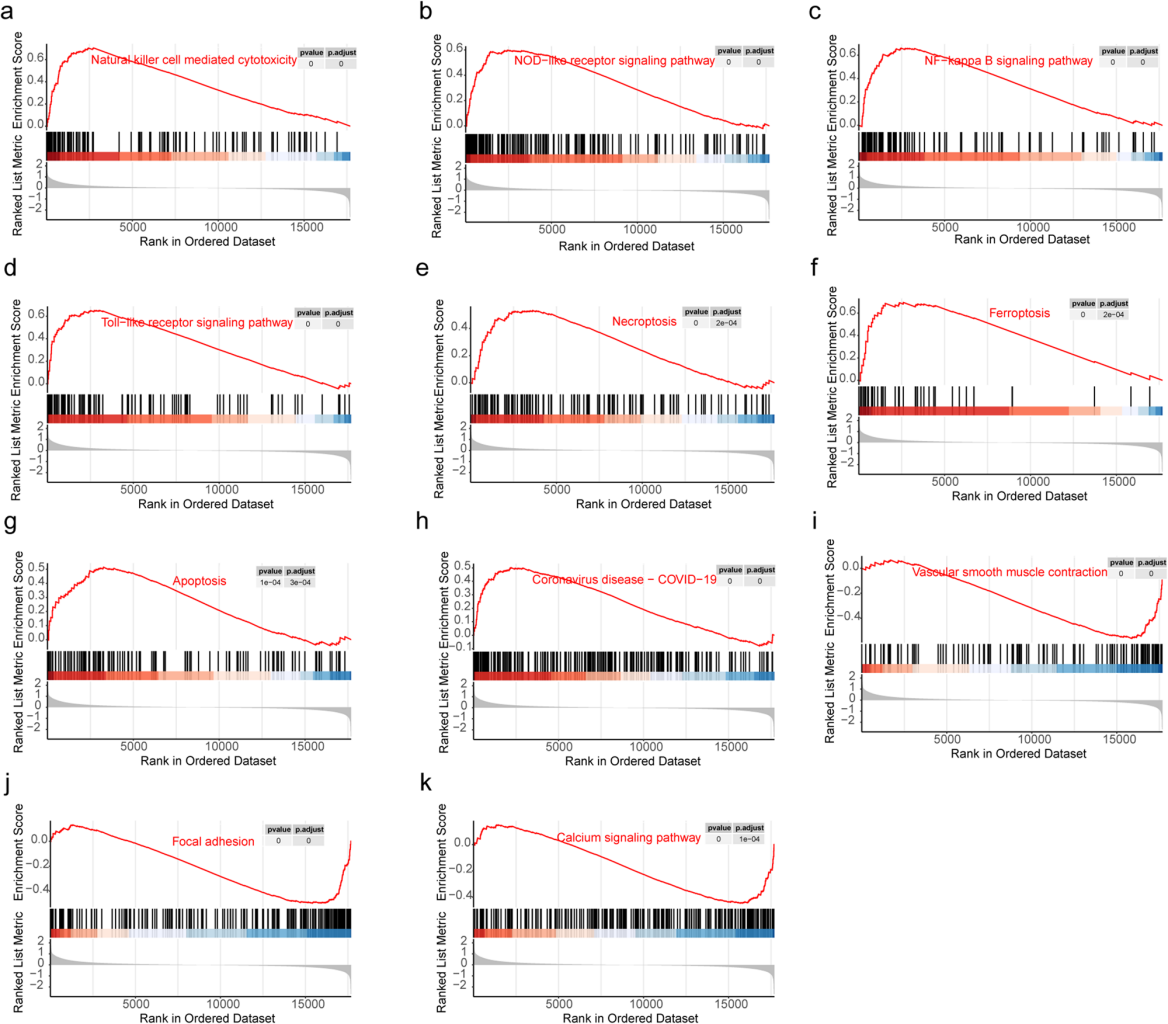
Gene set enrichment analysis (GSEA) comparing atheromatous plaques in PyroptosisScoreH and PyroptosisScoreL clusters. KEGG canonical pathways were used as a priori information for the GSEA. (**a**) NK cell-mediated cytotoxicity. (**b**) NOD-like receptor signaling pathway. (**c**) NF-kappa B signaling pathway. (**d**) Toll-like receptor signaling pathway. (**e**) Necroptosis. (**f**) Ferroptosis. (**g**) Apoptosis. (**h**) Covid-19 pathway. (**i**) Vascular smooth muscle contraction. (**j**) Focal adhesion. (**k**) Calcium signaling pathway.

We identified 266 DEGs (152 upregulated genes and 114 downregulated ones) when comparing high and low pyroptosis score clusters (Supplementary Table S4). We subsequently explored the DEGs’ functions with GO and KEGG analyses. Upregulated genes were enriched in biological processes (BP) involving regulation of leukocyte activation, adaptive immune response, and leukocyte mediated immunity, while downregulated genes were enriched in BP involving muscle system process and muscle contraction (Fig. 5a). Upregulated genes were enriched in molecular functions (MF) related to the tertiary granule, tertiary granule membrane and plasma lipoprotein particle, while downregulated genes were enriched in contractile fiber, myofibril and sarcomere. Upregulated genes were enriched in cellular components (CC) involving proteoglycan binding and endopeptidase activity, while most downregulated genes were enriched in structural constituents of muscle. KEGG analysis (Fig. 5b) revealed that upregulated genes were enriched in terms such as cholesterol metabolism, antigen processing, osteoclast differentiation, etc. Downregulated genes were mainly involved in focal adhesion, calcium signaling pathway, and vascular smooth muscle contraction. We compared immune-related pathway enrichment scores between the PyroptosisScoreH cluster and the PyroptosisScoreL cluster, as shown in Fig. 5c. All immune-related pathways but Type_II_IFN_Reponse were significantly higher in the PyroptosisScoreH compared to the PyroptosisScoreL cluster. Similar results were obtained comparing atheromatous plaques in different vascular beds (Supplementary Fig. S3b). GSEA analysis was conducted with FDR < 0.05 within PyroptosisScoreH and PyroptosisScoreL samples and as a result 119 terms were annotated (86 activated and 33 suppressed). Selected pathways were extracted and are displayed in Fig. 6a–k, while summary image and other pathways are shown in Supplementary Figs. S4 and S5a–t. Activated pathways were mainly associated with inflammation, immunity, viral infection, and cell death. Inhibited pathways were mainly related to smooth muscle cell function, extracellular matrix, energy metabolism, and cell connectivity.

### Identification key pyroptosis related genes and correlation of these genes with immune and stromal cells

**Figure 7:**
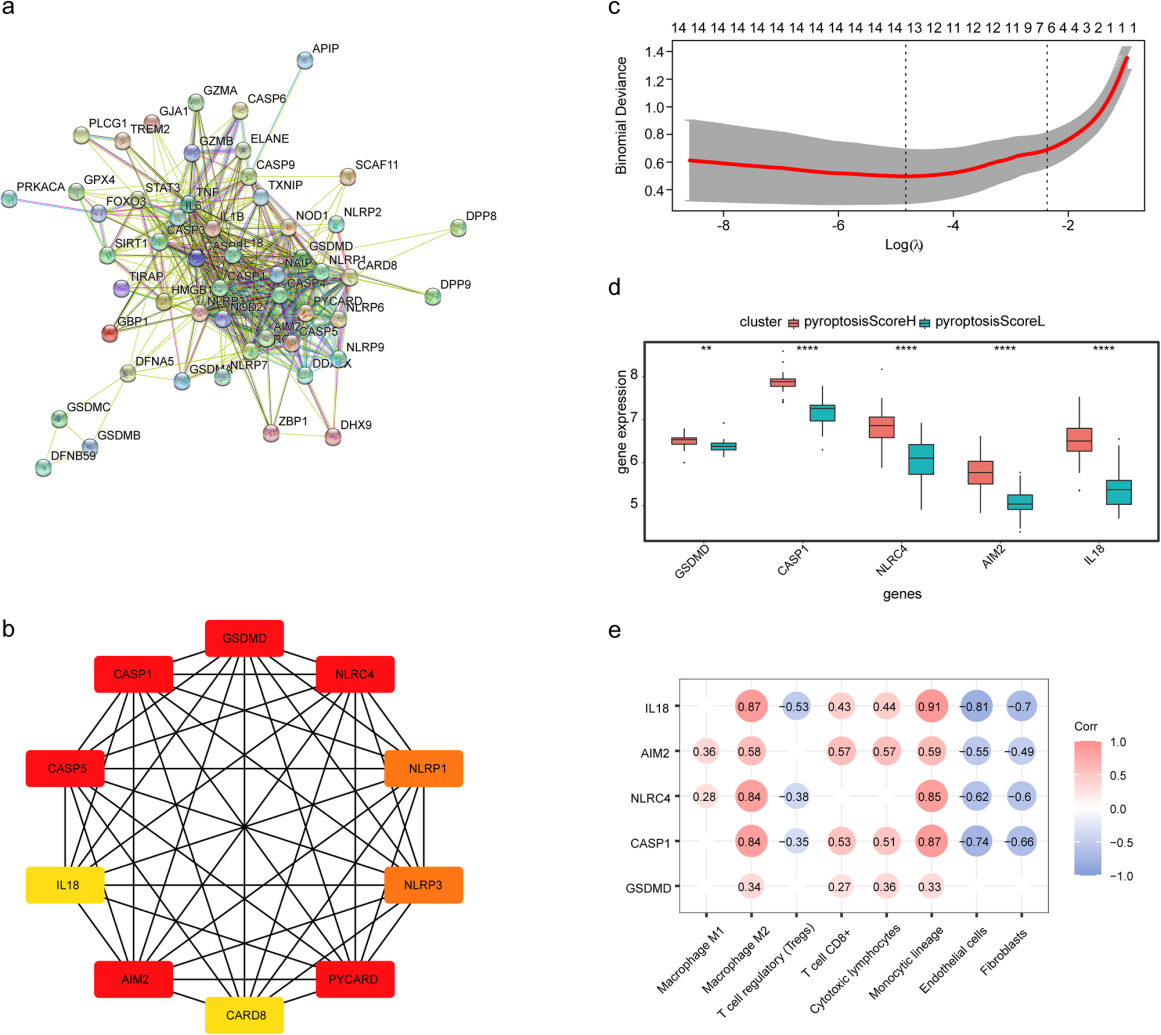
Hub genes and signature genes of pyroptosis and their correlations with immune and stromal cells. (**a**) PPI network of 50 pyroptosis-related genes in the PyroptosisScoreH cluster. (**b**) Ten hub genes in the PyroptosisScoreH cluster. (**c**) Signature gene selection by LASSO regression. (**d**) Comparison of expression levels of five genes (identified as both hub and signature genes) in the PyroptosisScoreH cluster and in the PyroptosisScoreL cluster. (**e**) Spearman correlation coefficients obtained from the correlations of GSDMD, CASP1, NLRC4, AIM2, and IL18 with the relative proportions of different types of immune cells.

Relationships among pyroptosis-related genes were analyzed via the STRING database in order to construct a PPI network (Fig. 7a). Ten hub genes (Supplementary Table S5) were identified using the MCODE and cytoHubba plugins of the Cytoscape software (Fig. 7b). 28 genes correlated with pyroptosis were selected by Spearman analysis among 52 pyroptosis-related genes. LASSO regression analysis was applied to these 28 genes and identified 13signature genes (Supplementary Table S6 and Fig. 7c). Genes identified in both the hub and signature sets (Gasdermin D (GSDMD), Cysteine-dependent aspartate-specific proteases-1 (CASP1), NLR Family CARD Domain Containing 4 (NLRC4), Absent In Melanoma 2 (AIM2), and Interleukin 18 (IL18)) were considered to be the key genes related to pyroptosis. We evaluated the expression of each identified genes and found that expression levels were significantly higher in the PyroptosisScoreH cluster (Fig. 7d). The correlation between these five genes and immune and stromal cells is shown in Fig. 7e. M1 macrophages were positively correlated with AIM2 and NLRC4. CD8 + T cells and cytotoxic lymphocytes were positively correlated with IL8, AIM2, CASP1, and GSDMD. M2 macrophages and the monocytic lineage were positively correlated with all five genes. T-regs were negatively correlated with IL8, NLRC4, and CASP1, while endothelial cells and fibroblasts were negatively correlated with all genes but GSDMD. Notably, the expressions of all five genes were significantly higher in carotid plaques than in femoral plaques and there no significantly differences between normal carotid arteries and normal femoral arteries (Supplementary Fig. S3c,d).

### Validation of expression levels of key pyroptosis related genes in vitro

**Figure 8:**
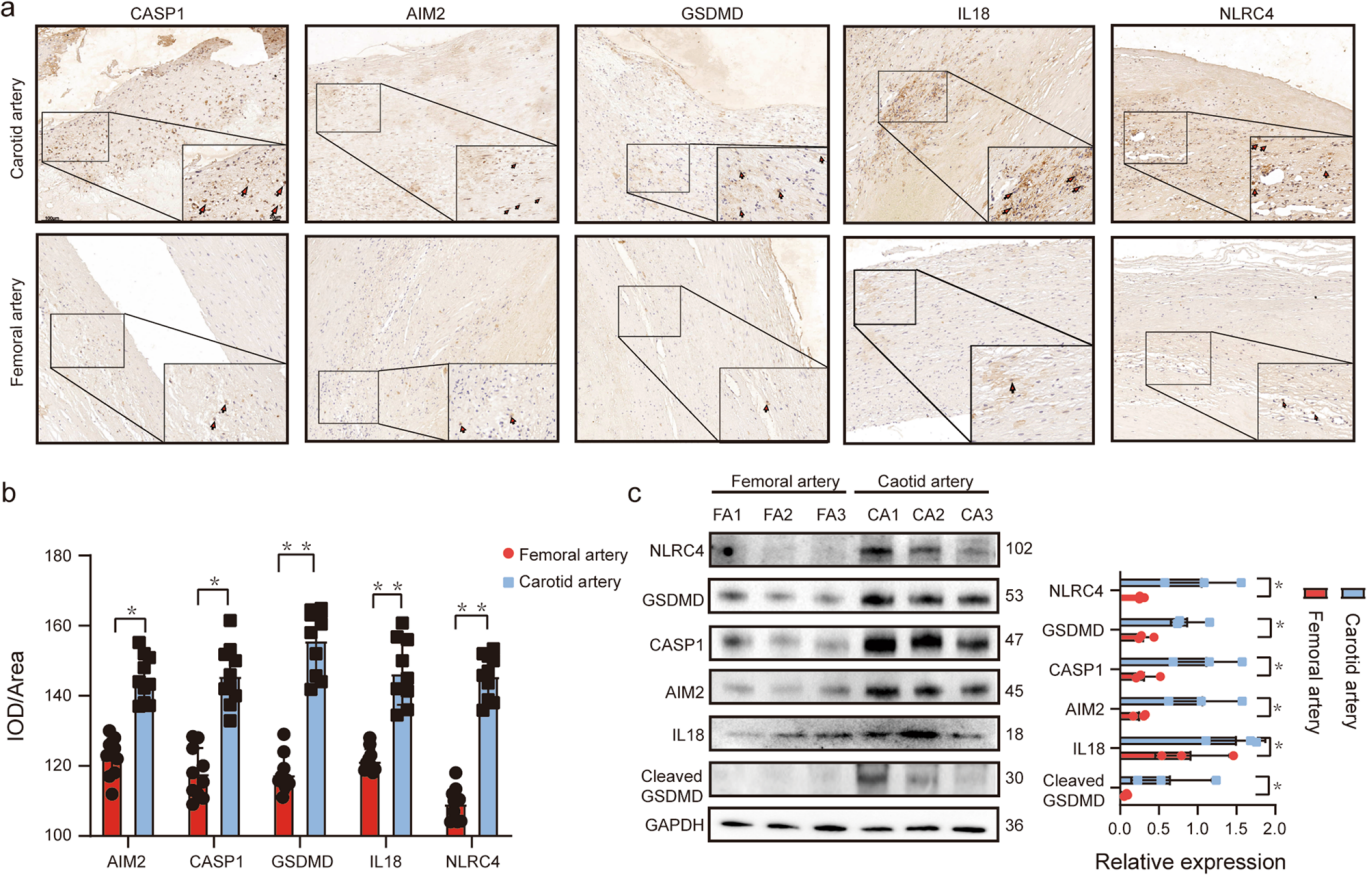
Five key pyroptosis-related genes expression in human advanced carotid plaques (n = 10) and femoral plaques(n = 10). (**a**) Representative images of immunohistochemistry staining of five key pyroptosis-related genes expression; Scale bars, 100 μm. Representative immunohistochemistry staining tracked by red arrows; Scale bars, 20 μm. (**b**) Quantification of the area positive for GSDMD, CASP1, NLRC4, AIM2 and IL18. The expression of these five genes were significantly increased in the site of severe lesions in advanced carotid plaques. IOD/Area: integrated optic density of sections stained in yellow–brown divided by total tissue areas. (**c**) Western blot analysis and corresponding quantification of five key pyroptosis-related genes expression. Representative blot from three independent experiments performed with n = 3 patients per experiment, and quantification of protein expression relative to GAPDH. Original blots are presented in Supplementary Information Fig. 1. *p < 0.05, ***p < 0.001.

According to the results of our bioinformatics analysis, compared with the advanced femoral plaques, the advanced carotid plaques have a higher degree of pyroptosis. Thus, we took further experiments, immunochemistry and immunoblotting, to validate our findings. Patient clinical characteristics and data, including age, gender, body mass index (BMI), hypertension, diabetes mellitus, dyslipidemia, smoking status, levels of C-reactive protein (CRP) and interleukin-6 (IL-6), and ischemic symptoms within 6 months before surgery, were collected retrospectively. These variables were compared between different groups for analysis. The results presented in Supplementary Table S7 showed that all clinical characteristics were not statistically different between the two groups, but the number of patients suffered from recent stroke or TIA was higher than those with lower limb ischemia. It might indicate the different plaques stability between carotid arteries and femoral arteries, which the former one was prone to rupture. As shown in Fig. 8a,b, we used IHC to preliminarily detect the expression of GSDMD, CASP1, NLRC4, AIM2 and IL18 in advanced atherosclerosis plaques of patients. The positive areas of these genes were significantly higher in the CEA group than the FEA group. The western blot results further confirmed these results (Fig. 8c). Activation of Caspase-1 usually represents the presence of pyroptosis state. We also detected the active caspase-1 to evaluated the degree of pyroptosis between the CEA group and the FEA group. The result implicated that the high level pyroptosis was associated with the occurrence of atherosclerosis in carotid arteries.

## Discussion

Atherosclerosis is a chronic inflammatory disease associated with innate and adaptive immunity^26,27^. Pyroptosis, an inflammation-dependent type of programmed cell death, is reported to be associated with atherosclerotic plaques^28,29^. Comparing pyroptosis and immune infiltration in plaques from different arterial regions could advance our understanding of mechanisms involved in atherosclerosis. In this study, we examined the pyroptosis status and immune infiltration of advanced atherosclerotic plaques from different vascular beds. We identified pyroptosis-related signaling pathways and five important pyroptosis-related genes and determined the relationship between these genes and different immune cells.

Our study provides preliminary evidence that plaque pyroptosis and immune infiltration are more severe in carotid artery than in femoral artery in advanced atherosclerosis. This may account for the more unstable plaques and the higher incidence of ischemia in patients with advanced carotid atherosclerosis compared those with advanced femoral atherosclerosis. And the finding also indicates that we should pay more attention to the degree of plaque pyroptosis in patients with advanced atherosclerosis, especially in carotid plaques, because it is more likely to lead to fatal complications such as stroke.

Our findings provide an important basis for further studies on the relationship between pyroptosis-related genes and immunity in atherosclerosis patients.

Using the GSE100927 dataset, we identified significant differences in gene expression in advanced atherosclerotic plaques from different vascular beds. After extracting and analyzing pyroptosis-related genes, we found that the expression of these genes is dependent on the vascular bed region. We used ssGSEA to calculate pyroptosis scores of samples, and grouped the plaques accordingly into a PyroptosisScoreH cluster and a PyroptosisScoreL cluster. We subsequently determined a positive correlation between immune scores and pyroptosis scores. The relationship between pyroptosis and immunity is consistent with existing literature^30,31^, which observed the release of cytokines, such as interleukin-1β (IL-1β) and interleukin-18(IL-18), during pyroptosis.

Using several methods, we directly compared the profiles of various types of immune cells and stromal cells in the PyroptosisScoreH and PyroptosisScoreL clusters, and found that advanced atherosclerotic plaques were mainly colonized by macrophages and lymphocytes. This is consistent with previous research showing that T cells and macrophages represent the largest population of leukocytes in atherosclerotic plaques^32,33^. We also found that in the PyroptosisScoreH cluster the proportion of Gamma delta T cells, CD8 + T cells, myeloid dendritic cells, and Cytotoxic lymphocytes was significantly higher than in the PyroptosisScoreL cluster while the trend in M2 macrophages was upregulation in low pyroptosis group. Pyroptosis was positively correlated with gamma delta T cells, CD8 + T cells (cytotoxic lymphocytes), macrophages and myeloid dendritic cells in all samples, and it was negatively correlated with NK cells, T-regs, fibroblasts, and endothelial cells (ECs). Previous studies have reported macrophage pyroptosis plays a significant role in the formation, rupture, and immuno-inflammatory response of atherosclerotic vulnerable plaques^34^, and symptomatic plaques have a higher percentage of M1 macrophages^11^. A recently published article using scRNA-seq data also showed that human femoral plaques exhibited noninflammatory foam cell-like macrophages compared with human carotid plaques^35^, which indicated distinct macrophage phenotypic and transcriptional profiles in different arterial territories.

Gasdermin-mediated pyroptosis is a mechanism that kills cytotoxic lymphocytes, mainly cytotoxic T lymphocytes (CTL or CD8 + T cells) and natural killer (NK) cells, that may enhance anti-tumor immune responses^36^. This is consistent with our findings that cytotoxic lymphocytes are positively correlated with pyroptosis in plaques of advanced atherosclerosis, although pyroptosis was negatively correlated with NK cells. This negative correlation could be due to the low number of NK cells in advanced plaques and the algorithms used, in which pyroptosis was positively related to NK cells in MCP-counter.

DCs are the most important antigen presenting cell-type and play a key role in initiating adaptive immune responses. A recent study has shown that pyroptosis of DCs has a crucial role in infectious diseases^37^. Considering the close relationship between infectious diseases and atherosclerosis^38^ and the positive correlation between pyroptosis and DCs observed in our research, the suppression of pyroptosis in dendritic cells could provide new directions for treatment of atherosclerosis.

Gamma delta T cells are mainly localized in mucosa-associated lymphoid tissues, and their function, which involves a non-specific cell-killing effect, falls between innate and adaptive immunity. So far, no study has proven the relationship of gamma delta T cells with pyroptosis, but their proliferation in adipose tissue is known to promote macrophage accumulation, inflammation, and insulin resistance in obese mice fed with a high fat diet^39^, along with the secretion and release of perforin, granzyme, and interferon-γ. These secretions are also involved in the processes of pyroptosis, providing clues into the connection between gamma delta T cells and pyroptosis in atherosclerosis.

EC pyroptosis contributes to the formation and progression of atherosclerosis promoting the recruitment of monocytes into the vascular intima during early-stage disease^40^. Recruited monocytes engulf a large amount of circulating low-density lipoprotein (LDL), leading to endothelial cell damage and starting a detrimental cycle. A recent clinical study revealed that both CD8^+^T cells and their cytotoxic effector molecules enriched in atherosclerotic lesions and caused endothelial cell death^41^. It is consisting with our result that CD8^+^T cells is negatively correlated to the EC. This mechanism explains the negative correlation between pyroptosis and proportion of ECs in advanced atherosclerotic plaques. T-regs could increase the stability of plaques by reducing the migratory ability of DCs and inhibiting the adhesion of DCs to ECs^42^. A lower fraction of T-regs was found in advanced carotid atherosclerotic plaques compared to early plaques^43^. In an Optical Coherence Tomography (OCT) study in Acute Coronary Syndromes^44^, an inverse correlation between CD4^+^CD28^null^/Treg ratio and cap-thickness is reported, and this means the decrease in Treg frequency was accompanied by cap-thickness thinning, predisposing atherosclerotic plaque to rupture. In another study^45^, decreased local Treg numbers tend to exhibit higher perivascular fat attenuation index (FAI) values, which increased FAI values were associated with vulnerable plaque components. Pyroptosis, however, decreases the stability of advanced atherosclerotic plaques^34^. It is therefore likely that T-regs are negatively correlated with pyroptosis, as observed in our study.

Because of the highly proinflammatory features and critical role of pyroptosis in the atherosclerotic immune response, we performed GSEA analysis to identify affected pathways. Suppressed pathways were mainly enriched in vascular smooth muscle contraction, focal adhesion, and calcium signaling pathway, which are closely related to plaque stability. The PyroptosisScoreH cluster was markedly enriched in immune full-activation pathways such as NK cell-mediated cytotoxicity, NOD-like receptor signaling pathway, cytokine-cytokine receptor interactions, NF-kappa B signaling pathway, toll-like receptor signaling pathway, and chemokine signaling pathway, all of which are involved in the pyroptosis process during atherosclerosis^30,46–48^. Notably, pathways related to necroptosis, ferroptosis, and apoptosis were also activated, indicating different programmed cell death pathways may happen in synergy in the progression of atherosclerosis^49^. The COVID-19 pathway was also enriched in the PyroptosisScoreH cluster, indicating SARS-CoV-2 infection might promote the progression of atherosclerosis and increase the vulnerability to it through the induction of pyroptosis, which in turn could cause severe strokes. The results are consistent with published studies^50,51^ that found COVID-19 patients had an increased risk of ischemic stroke. It may be that pyroptosis in atheromatous plaques contributes to higher stroke risks. COVID-19 patients who experience stroke have a worse prognosis than patients who do not experience stroke^52^. Altogether, in the context of the global COVID-19 pandemic, targeting pyroptosis is crucial for the effective treatment of atherosclerosis.

We identified a set of genes (*GSDMD, CASP1, NLRC4, AIM2,* and *IL18*) closely related to atherosclerosis, particularly in atherosclerotic lesions with high pyroptosis scores. Prior research has found these genes to be involved in the pyroptosis process in atherosclerotic plaques^29–31^. This is consistent with our findings that most of these genes are positively correlated with immune cells (macrophages and cytotoxic lymphocytes) that promote pyroptosis. Caspases-1 (cysteine-dependent aspartate-specific proteases-1) is activated by inflammasomes, such as nucleotide-binding oligomerization domain-like receptors (NLP, e.g., NLRP3, NLRC4) and Non-NLR absent in melanoma-2 (AIM2). Caspase-1 cleaves gasdermin D (GSDMD), opening pores that act as direct conduits for the transport of IL-1β and IL-18, and cleaves pro-IL-1β and pro-IL-18 into their active forms, leading to pyroptosis in plaques^34^. Excessive repression of inflammation leads to atherosclerotic plaque instability^53^, while overexpression of IL-18-binding protein makes plaques more stable^54^. All available evidence suggests GSDMD, CASP1, NLRC4, AIM2, and IL18 could be therapeutic targets for the treatment of atherosclerotic plaques with elevated pyroptosis, and balancing the degree of pyroptosis in plaques may be the principle of treatment.

It is important to note that, in this study, all but one of the carotid plaques were classified as PyroptosisScoreH and the majority of femoral plaques were classified as PyroptosisScoreL. Given that all plaques were in an advanced state, identifying the differences between them might contribute to a better understanding of atherosclerosis. We believe there are two possible reasons to explain the observed differences: one, the anatomical morphology of blood vessels in different areas are different. The curvature of the carotid artery is smaller than that of the femoral artery and experiences less shear stress^55^. A low shear force could promote EC pyroptosis, causing damage to the vascular wall and tissue remodelling, hence leading to the onset and development of atherosclerosis^56,57^. The second reason could be that the heterogeneity of active genes in different vascular beds leads to different sensitivities to the same stimuli, in turn leading to different disease phenotypes^7^. This finding was also consistent with Wang et al.’s study which showed that pathways related to necroptosis, pyroptosis and ferroptosis are enriched in carotid compared to femoral plaques. They found that the proportion of inflammatory macrophages was significantly higher in the carotid artery than in the femoral artery^58^.

We recognize some limitations in our study. First, the plaques in the dataset GSE100927 did not include pathological information, such as stability, vulnerability, or calcification, therefore it was not possible to determine whether the differences in pyroptosis scores are related to different pathological types. Second, our sample set was small, which may have reduced the precision of data analysis and its results. As more sequencing results become available, more samples can be included for validation. Finally, our study lacked of plasma/serum data supporting NLRP3 activation/inflammation, which were essential to confirm the presence of systemic pyroptosis. Besides, we performed experimental validation in vitro, the number of cases was small and more patients were needed to recruit to validate our findings and further experiments in vivo are necessary to verify the underling molecular mechanism.

## Conclusion

To the best of our knowledge, our work is the first to apply bioinformatics methods to the investigation of the role of pyroptosis in advanced atherosclerotic plaques based on different vascular beds and to explore immune infiltration in plaques with different pyroptosis states. Our study provides valuable insight into atherosclerosis in different vascular beds: pyroptosis appears to have a bigger role in the formation of carotid artery plaques than of femoral artery plaques, indicating that carotid plaques are more likely to cause fatal outcomes. In conclusion, the findings of this study, which encompass pyroptosis-related immune cells, pyroptosis-related genes, and pyroptosis-related pathways, establish a theoretical foundation for researchers and clinicians to explore the potential of targeting pyroptosis in the management of advanced atherosclerosis, especially in carotid arteries.

### Supplementary Information

Supplementary Figures.

Supplementary Information.

Supplementary Table S1.

Supplementary Table S2.

Supplementary Table S3.

Supplementary Table S4.

Supplementary Table S5.

Supplementary Table S6.

Supplementary Table S7.

## Data Availability

Microarray data were downloaded from the GEO database (http://www.ncbi.nlm.nih.gov/geo/) and contained 29 atheromatous carotid plaques and 26 atheromatous femoral plaques.
