# Supplementary material for: Identification of key pyroptosis-related genes and microimmune environment among peripheral arterial beds in atherosclerotic arteries
Source: Sci Rep. 2024 Jan 2;14:233. doi: 10.1038/s41598-023-50689-x (PMC10761966; doi:10.1038/s41598-023-50689-x)
Supplement: Supplementary file 2 — Supplementary Information. [file 41598_2023_50689_MOESM2_ESM.docx]

Identification of key pyroptosis-related genes and microimmune environment among peripheral arterial beds in atherosclerotic arteries

Jing-Wen Liu^1,2†^, Zhao-Hua Zhang^1,2†^, Xiao-Shuo Lv^2,3†^, Ming-Yuan Xu^1,2^, Bin Ni^1,2^, Bin He^2^, Feng Wang^3^, Jie Chen^2^, Jian-Bin Zhang^2^, Zhi-Dong Ye^2^, Peng Liu^1,2*^, Jian-Yan Wen^2*^

**
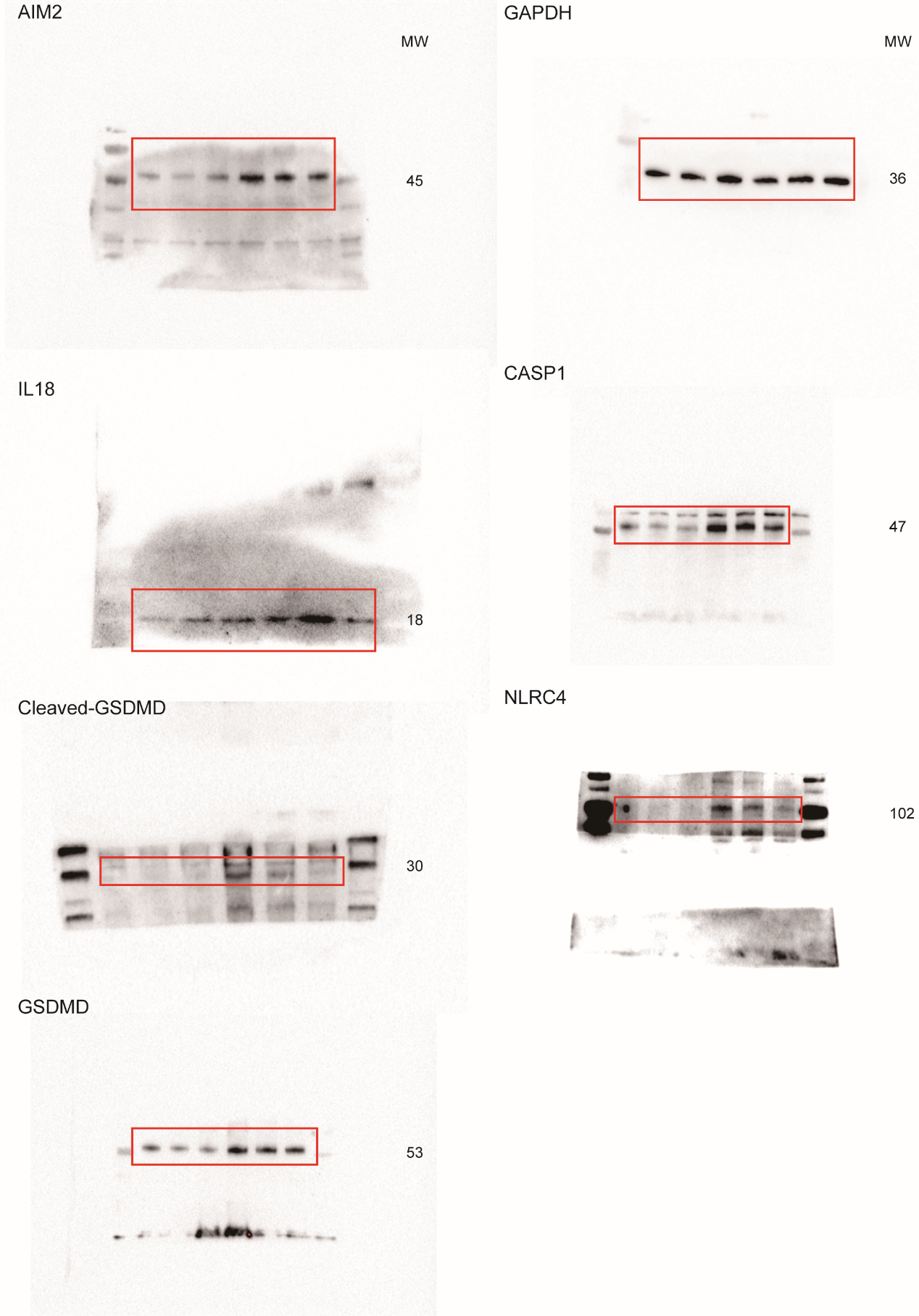
**

**Figure.1.** Original blots of five key pyroptosis-related genes expression.

**Supplementary Figures**

**Supplementary Figure.S****1.** Intra-sample data repeatability test for GSE100927 using Spearman’s correlation analysis and PCA. **(a)** Spearman’s correlation analysis of samples from the GSE100927 dataset. The blue-to-red scale indicates coefficients from 0.86 to 1. **(b)** Samples from the GSE100927 dataset were analyzed using PCA. PC1 and PC2 are represented respectively on the X-axis and Y-axis. PCA, principal component analysis; PC1, principal component 1; PC2, principal component 2.

**Supplementary Figure.S2.** A bar plot showing the proportions of 22 immune cells in the PyroptosisScoreH and PyroptosisScoreL clusters.

**Supplementary Figure.S3.** Immune cell abundance, immune-related pathway enrichment, and hub/signature gene expression in different clusters and groups. **(a)** The relative abundance of 4 types of immune cells in the PyroptosisScoreH and PyroptosisScoreL clusters; **(b)** Comparison of the enrichment scores of 13 immune-related pathways between carotid plaques and femoral plaques. (NS: p > 0.05, *p < = 0.01, ***p≤ 0.001, ****p≤ 0.0001); **(c)** Comparison of the expression of five key genes related to pyroptosis between carotid plaques and femoral plaques; **(d)** Comparison of the expression of five key genes related to pyroptosis between normal carotid arteries and normal femoral arteries.

**Supplementary Figure.S4.** Overview of up-regulated and down-regulated pathways of interest (a longer bar indicates more enriched genes; red represents an activated pathway, green a suppressed pathway, with increasing color-depth indicating more obvious differences).

**Supplementary Figure.S5.** Gene set enrichment analysis (GSEA) comparing the PyroptosisScoreH and PyroptosisScoreL clusters. **(a)** Th1 and Th2 cell differentiation, **(b)** B cell receptor signaling pathway, **(c)** Chemokine signaling pathway, **(d)** Osteoclast differentiation, **(e)** Cholesterol metabolism, **(f)** Cytokine-cytokine receptor interaction, **(g)** PPAR signaling pathway, **(h)** Phagosome, **(i)** T cell receptor signaling pathway, **(j)** TNF signaling pathway, **(k)** Leukocyte transendothelial migration, **(l)** JAK-STAT signaling pathway, **(m)** PI3K-Akt signaling pathway, **(n)** MAPK signaling pathway, **(o)** cAMP signaling pathway, **(p)** Tight junction, **(q)** Cardiac muscle contraction, **(r)** Proteoglycans in cancer, **(s)** Signaling pathways regulating pluripotency of stem cells and **(t)** Apelin signaling pathway.

**Supplementary Table**

**Supplementary Table S1.** Immune pathway-related genes.

**Supplementary Table S2.** Primary antibodies used in Western Blot.

**Supplementary Table S3.** Lists of 52 pyroptosis-related genes.

**Supplementary Table S4.** DEGs in the PyroptosisScoreH cluster and PyroptosisScoreL cluster.

**Supplementary Table S5.** Lists of 10 hub genes of pyroptosis-related genes.

**Supplementary Table S6.** Lists of 13 signature genes of pyroptosis.

**Supplementary Table S7.** Patient demographic data.
